# A comparison of different definitions of metabolic syndrome for the risks of atherosclerosis and diabetes

**DOI:** 10.1186/s13098-018-0358-x

**Published:** 2018-07-16

**Authors:** Sung-Sheng Tsai, Yun-Yi Chu, Szu-Tah Chen, Pao-Hsien Chu

**Affiliations:** 1 Division of Endocrinology and Metabolism, Chang Gung Memorial Hospital, Chang Gung University College of Medicine, Taipei, Taiwan; 2 Department of Materials Science and Engineering, Chiao-Tung University, Hsinchu, Taiwan; 3 Department of Cardiology, Chang Gung Memorial Hospital, College of Medicine, Chang Gung University, 199 Tun-Hwa North Road, Taipei, 105 Taiwan; 4 Healthcare Center, Chang Gung Memorial Hospital, Chang Gung University College of Medicine, Taipei, Taiwan

**Keywords:** Metabolic syndrome, Diabetes, Atherosclerosis, Arterial stiffness, Cardiovascular disease

## Abstract

**Background:**

The reported outcomes of the metabolic syndrome (MetS), containing atherosclerotic cardiovascular disease and diabetes, vary according to the definitions used. This study was designed to compare the performance of the Adult Treatment Panel III/American Heart Association/National Heart, Lung, and Blood Institute (ATP III/AHA/NHLBI) and International Diabetes Federation (IDF) criteria for the risks of atherosclerosis and diabetes.

**Methods:**

We sifted subjects from a self-paid Health examination program from 1999 to 2015 in this cross-sectional population-based study. On the basis of the ATP III/AHA/NHLBI and IDF criteria, the MetS diagnosis and scores were concluded. A brachial-ankle pulse wave velocity (baPWV) more than or equal to 1400 cm/s indicated more severe arterial stiffness, and a high fasting glucose level more than or equal to 6.99 mmol/L or postprandial glucose level more than or equal to 11.10 mmol/L indicated diabetic-level hyperglycemia. Comparisons of the areas under receiver operating characteristic curves (AUC–ROC) for both MetS scores to correlate with a higher baPWV and diabetic-level hyperglycemia were evaluated.

**Results:**

In the 26,735 enrolled subjects with an average age of 55 (± 12) years, 6633 and 7388 (24.8% vs. 27.6%, *p* < 0.001) were classified as having MetS on the basis of the ATP III/AHA/NHLBI and IDF criteria, respectively. The AUC–ROC for the ATP III/AHA/NHLBI-MetS score were higher than those for the IDF-MetS score (0.685 vs. 0.595 to correlate with a higher baPWV, *p* < 0.001; 0.791 vs. 0.665 to correlate with diabetic-level hyperglycemia, *p* < 0.001).

**Conclusions:**

To the best of our knowledge, this is the first study to demonstrate that through a holistic approach, the performance of the ATP III/AHA/NHLBI-MetS score for the risks of atherosclerosis and diabetes was superior to the IDF-MetS score for Asians.

## Background

Throughout the world, the prevalence of the metabolic syndrome (MetS) is rising [1, 2]. MetS is set as a group of several characteristics including a broadened waist circumference (WC) caused by hypernutrition, a sedentary lifestyle, and sequential abdominal fat accumulation [1, 2]. Dr. Reaven first described the term “syndrome X” in 1988 with simultaneously grouped risk factors [3]. Syndrome X, later termed MetS, is recognized as a multiplex risk factor for type 2 diabetes and atherosclerotic cardiovascular disease (ASCVD) due to high correlations with insulin resistance. MetS is currently described as the grouping of abdominal obesity, insulin resistance, elevated blood pressure (BP), and obesity-related dyslipidemia, while also highly correlated with other clinical disorders such as nonalcoholic fatty liver disease, a prothrombotic or proinflammatory state, and reproductive problems [4–6]. Previous studies have revealed that MetS is correlated with unfavorable clinical outcomes, including a fivefold increased risk of new diagnosed type 2 diabetes, and an approximate twofold increased risk of ASCVD [5].

Currently, there are many different MetS definitions, as the MetS is a combination of different unfavorable conditions with the joint pathophysiology of abdominal obesity and insulin resistance, and not just one single disease [5]. Moreover, the prevalence and outcome data of the MetS depend on these criteria used and vary [7, 8]. Hence, it is necessary to find a champion criterion for MetS evaluation. Even though some different MetS criteria were modulated to the one in 2009 [9], the National Cholesterol Education Program-Adult Treatment Panel III (ATP III), adjusted ATP III by the American Heart Association and the National Heart, Lung, and Blood Institute (ATP III/AHA/NHLBI) [10] and the International Diabetes Federation (IDF) [11] are still the most commonly used criteria throughout the world [12].

Atherosclerosis-related arterial stiffness is examined by pulse wave velocity (PWV), which is highly associated with ASCVD and has been well-studied as a powerful risk factor for coronary artery disease [13] and all-cause death [14] in general populations. Moreover, the PWV is also a strong risk factor for death, beyond systolic blood pressure (SBP), among patients with glucose intolerance or diabetes [15]. However, the associations between the different MetS criteria and clinical outcomes have not yet been elucidated. The purpose of this current study was to objectively compare the performance of ATP III/AHA/NHLBI- and IDF-MetS scores for the risks of atherosclerosis and diabetes through a holistic approach in a general population.

## Methods

### Data source and study population

**Fig. 1:**
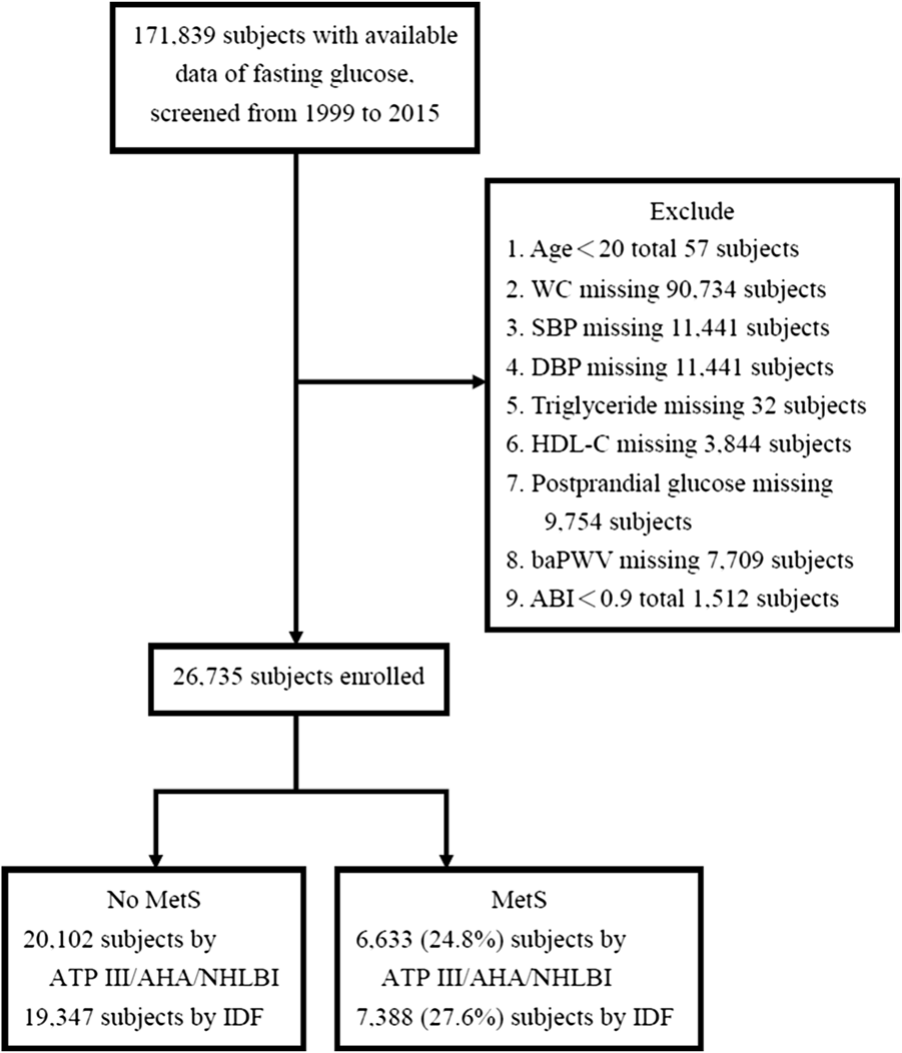
Identification of the study groups. *ABI* ankle/brachial index, *baPWV* brachial-ankle pulse wave velocity, *DBP* diastolic blood pressure, *HDL-C* high-density lipoprotein cholesterol, *MetS* metabolic syndrome, *SBP* systolic blood pressure, *WC* waist circumference

Database from 1999 to 2015 from a Health Examination Program at one’s own expense at the Health Care Center of Taoyuan Chang Gung Memorial Hospital was gathered in the cross-sectional population-based study. In our recent report, the database and the selection criteria have been described previously to test the weight of different WC cut-off values of the MetS diagnosis for the risks of atherosclerosis and diabetes in a Chinese population [16]. Briefly, every subject was demanded to be asymptomatic for infection and to complete a uniform protocol, containing well-designed questionnaires revealing their family and personal lifestyle patterns and medical histories, as well as measurements of body weight, height, BP, WC, and brachial-ankle PWV/ankle-brachial index (baPWV/ABI), applied to estimate the severity of arterial stiffness. The inclusion guide was obtainable information of fasting plasma glucose, and 171,839 subjects in all were sifted. The exclusion guides were less than 20 years old, an ABI value less than 0.9, or any missing information of the other 4 MetS components, post-prandial plasma glucose or baPWV (Fig. 1). The included subjects were diagnosed as the MetS or not on the basis of the ATP III/AHA/NHLBI and IDF criteria.

All subjects were required to give their written informed consent. The Ethics Committee of the Institutional Review Board of Chang Gung Memorial Hospital (Approval Number: 102-4175B) approved the study, and we did on basis of the ethical principles of the Declaration of Helsinki.

### Outcome definitions

A baPWV more than or equal to 1400 cm/s was set as a higher level indicating more serious arterial stiffness [17]. A high fasting plasma glucose level more than or equal to 6.99 mmol/L or postprandial plasma glucose level more than or equal to 11.10 mmol/L was set as diabetic-level hyperglycemia indicating an increased risk of diabetes [18].

### MetS diagnosis and scores

On the basis of the 2005 ATP III/AHA/NHLBI [10] and 2005 IDF [11] criteria, the MetS diagnosis and scores were concluded. The former contains: (1) raised WC (≥ 102 cm for men or ≥ 88 cm for women); (2) elevated BP (≥ 130/85 mmHg); (3) raised fasting plasma glucose level (≥ 5.55 mmol/L); (4) decreased plasma high-density lipoprotein cholesterol level (HDL-C) (< 1.0 mmol/L for men or < 1.3 mmol/L for women); and (5) raised plasma triglyceride level (≥ 1.7 mmol/L). Any 3 of the above 5 components established the ATP III/AHA/NHLBI-MetS diagnosis. All components in the IDF definition were the same as in the ATP III/AHA/NHLBI except for the WC cutoff value (≥ 90 cm for men or ≥ 80 cm for women, considering abdominal obesity as its core component). Abdominal obesity plus any 2 of the other 4 components established the IDF-MetS diagnosis. Each component contributed one point to the ATP III/AHA/NHLBI- and IDF-MetS scores for each subject. For the IDF-MetS score, all subjects who did not meet the WC cutoff value were given a score of 0.

### Anthropometric and laboratory measurements

Brachial BP measured by an appropriately sized cuff was recorded in duplicate, and values were reported as a mean of two recordings. WC was measured at the midpoint between the iliac crest and lowest rib when a subject was in a standing position with feet 15 cm apart. All subjects underwent laboratory measurements, and after fasting overnight for 12 h, venous serum samples were gathered to test in the early morning. The biochemical measurements contained tests of fasting plasma glucose, HDL-C, and triglyceride at the only laboratory of our hospital. We supplied the uniform food and drink for every subject, and tested their post-prandial plasma glucose after 2 h.

### Measurements of baPWV

An automated machine (Colin VP-1000, Omron, Japan) were applied to achieve measurements of baPWV. Details were recorded in our previous report [19]. Briefly, all subjects were demanded not to consume any stimulant something, including cigarette, coffee or alcoholic drinks overnight. The special examination room was kept at a control atmospheric temperature. The technicians operating at our single center were well-disciplined and familiar with their work. Both baPWVs were computed as the distance/duration between both ankles and the right arm, and their average was represented as the last reported baPWV [16, 19, 20].

### Statistical analysis

We applied SPSS version 22.0 for Windows 7 (SPSS Inc., Chicago, USA) to analyze most data which were presented as the average (± SD) or frequency in last. A *p* value less than 0.05 was applied to determine statistical significance. We applied the Kolmogorov–Smirnov test to assess all variables for normal distribution. Moreover, the comparisons were performed using one-way analysis of variance for continuous variables and using 2-sample proportions test for the prevalence of the both MetS. We plotted receiver operator characteristic (ROC) curves for the ATP III/AHA/NHLBI- and IDF-MetS scores to correlate with a higher baPWV and diabetic-level hyperglycemia. Next we compared the areas under the ROC curves (AUC–ROC) for the performance by applying R software version 3.3.2 (R Foundation, Austria). The optimal cutoff points of both MetS scores were obtained by computing the equation: sensitivity − (1 − specificity), when the maximum values were reached, called the Youden index [21].

## Results

### Baseline characteristics of study subjects

In all, 26,735 adult subjects were concluded from 2006 to 2015 with an average age of 55 (± 12) years (range 20–101 years), WC of 87 (± 12) cm, SBP of 135 (± 20) mmHg, DBP of 81 (± 12) mmHg, fasting glucose level of 5.55 (± 1.55) mmol/L, post-prandial glucose level of 6.44 (± 3.00) mmol/L, HDL-C level of 1.32 (± 0.36) mmol/L, triglyceride level of 1.57 (± 1.13) mmol/L, and baPWV of 1506 (± 336) cm/s. Among them, 10,514 (39.3%) were female and 2738 (10.2%) suffered from diabetic-level hyperglycemia.

The number of individual components of both MetS definitions included: (1) 4158 (15.6%) for WC according to the ATP III/AHA/NHLBI-MetS and 12,602 (47.1%) according to the IDF-MetS; (2) 15,713 (58.8%) for BP; (3) 8223 (30.8%) for fasting glucose; (4) 8408 (31.4%) for triglyceride; and (5) 7751 (29.0%) for HDL-C. In all 26,735 subjects, 6633 (24.8%, 95% CI 24.3–25.3) with an average age of 57 (± 12) years and 2695 (41%) female were classified as having the ATP III/AHA/NHLBI-MetS compared to 7388 (27.6%, 95% CI 27.1–28.2) with an average age of 57 (± 12) years and 3107 (42%) female as having the IDF-MetS (24.8% vs. 27.6%, *p* < 0.001).

### Both MetS scores and atherosclerosis-related arterial stiffness

**Fig. 2:**
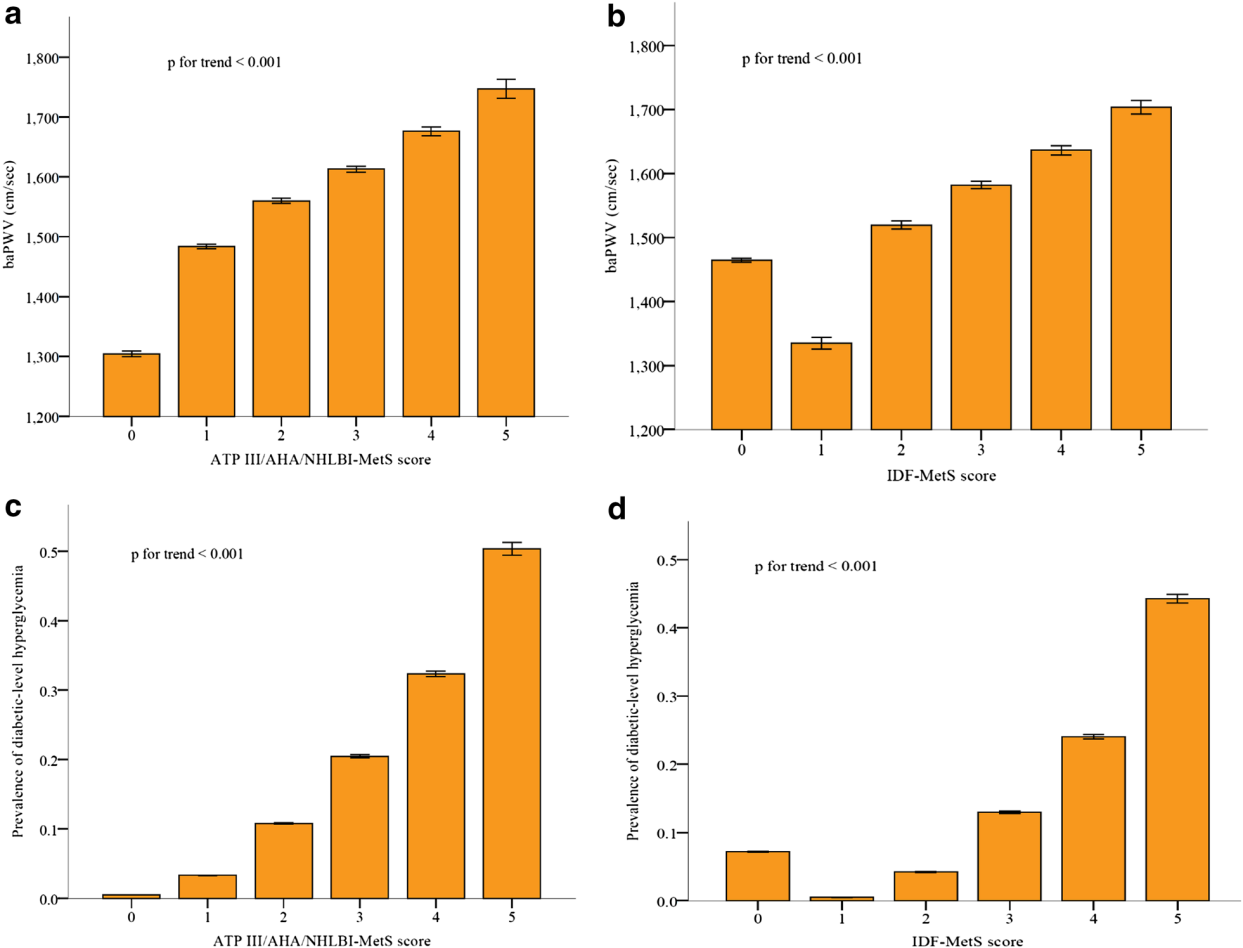
Associations of the baPWV and the prevalence of diabetic-level hyperglycemia and the (**a**, **c**) ATP III/AHA/NHLBI- and (**b**, **d**) IDF-MetS scores after adjusting for sex and age. All between-scores differences were significant (*p* < 0.001). *baPWV* brachial-ankle pulse wave velocity, *MetS* metabolic syndrome

After adjusting for sex and age, a stepwise raise in the baPWV corresponded to the ATP III/AHA/NHLBI-MetS score (*p* for trend < 0.001) (Fig. 2a). In addition, except for a score of 0 indicating no abdominal obesity, a stepwise raise in baPWV also corresponded to the IDF-MetS score after adjusting for sex and age (*p* for trend < 0.001) (Fig. 2b).

### Both MetS scores and diabetes risk

After adjusting for sex and age, a stepwise raise in the prevalence of diabetic-level hyperglycemia corresponded to the ATP III/AHA/NHLBI-MetS score (*p* for trend < 0.001) (Fig. 2c). In addition, except for a score of 0 indicating no abdominal obesity, a stepwise raise in the prevalence of diabetic-level hyperglycemia also corresponded to the IDF-MetS score after adjusting for sex and age (*p* for trend < 0.001) (Fig. 2d).

### Performance of both MetS scores

**Fig. 3:**
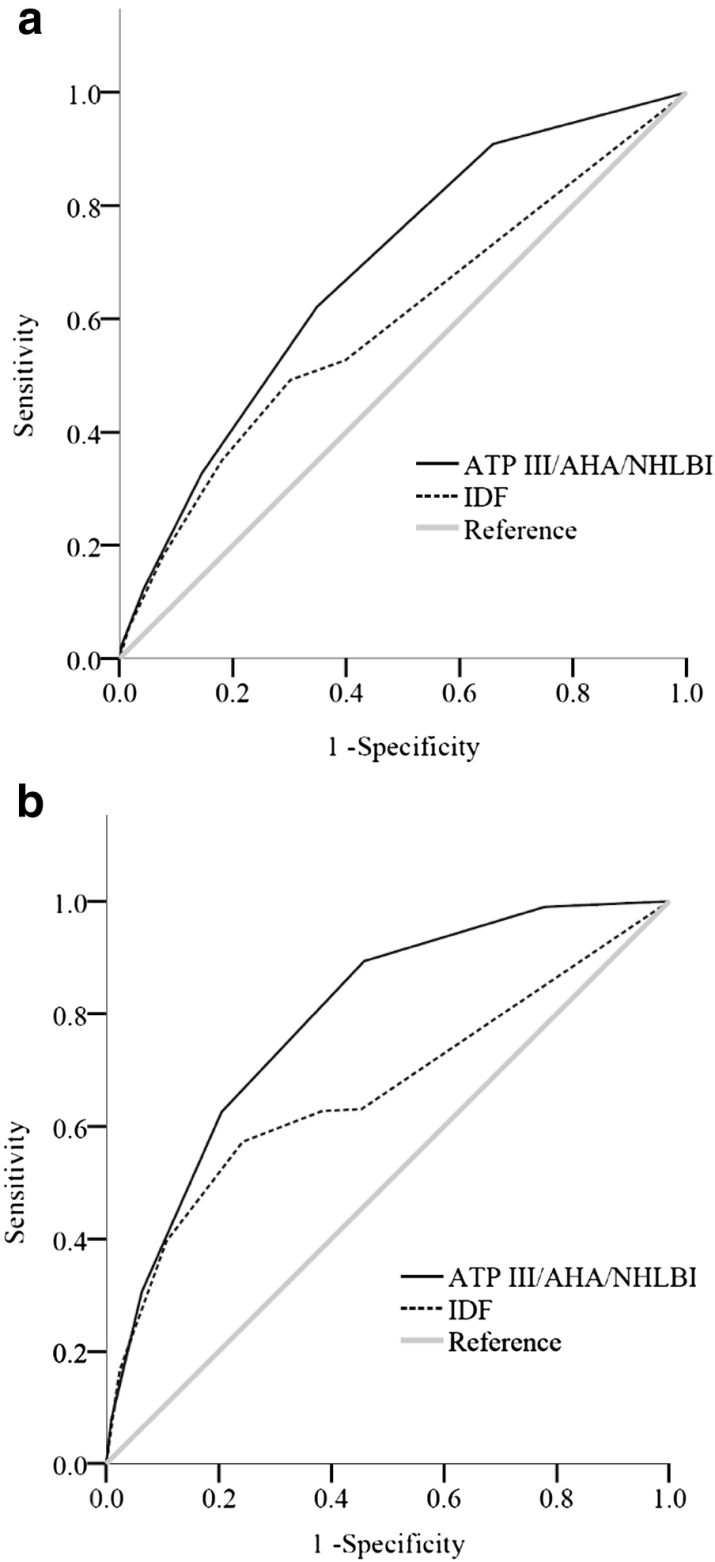
Receiver operating characteristic curves for the ATP III/AHA/NHLBI- and IDF-MetS scores to correlate with **a** a higher baPWV and **b** diabetic-level hyperglycemia. *baPWV* brachial-ankle pulse wave velocity, *MetS* metabolic syndrome

The AUC-ROC for the performance of the ATP III/AHA/NHLBI-MetS score to correlate with a higher baPWV was 0.685 compared to 0.595 for the IDF-MetS score (Fig. 3a). The AUC of the former was significantly higher than that of the latter (*p* < 0.001) (Table 1). There was no effect of gender because of the gender-specific WC cutoff values in both MetS criteria.

**Table 1:** Comparisons of the areas under receiver operating characteristic curves for the ATP III/AHA/NHLBI- and IDF-MetS scores to correlate with a higher baPWV and diabetic-level hyperglycemia

Outcome	Definitions	AUC	Lower	Upper	*p* value	Comparison *p*-value
Higher baPWV	ATP III/AHA/NHLBI	0.685	0.679	0.692	< 0.001	< 0.001
	IDF	0.595	0.588	0.601	< 0.001	
Diabetic-level hyperglycemia	ATP III/AHA/NHLBI	0.791	0.783	0.799	< 0.001	< 0.001
	IDF	0.665	0.653	0.678	< 0.001	

*AUC* area under the ROC curve, *baPWV* brachial-ankle pulse wave velocity, *MetS* metabolic syndrome

The AUC-ROC for the performance of the ATP III/AHA/NHLBI-MetS score to correlate with diabetic-level hyperglycemia was 0.791 compared to 0.665 for the IDF-MetS score (Fig. 3b). The AUC of the former was significantly higher than that of the latter (*p* < 0.001) (Table 1). There was no effect of gender because of the gender-specific WC cutoff values in both MetS criteria.

### Optimal cutoff points of both MetS scores

The optimal cutoff point of the ATP III/AHA/NHLBI-MetS score to correlate with a higher baPWV was more than or equal to two, with a better sensitivity from 32.7 to 62.2% (Table 2). Likewise, that of the IDF-MetS score was more than or equal to two, with a better sensitivity from 35.0 to 49.2%.

**Table 2:** Optimal cut-off points of the ATP III/AHA/NHLBI- and IDF-MetS scores to correlate with a higher baPWV and diabetic-level hyperglycemia

Higher baPWV	ATP III/AHA/NHLBI			IDF		
Cut-off point	Sensitivity	1-specificity	Youden index	Sensitivity	1-specificity	Youden index
≥ 0	1.000	1.000	0.000	1.000	1.000	0.000
≥ 1	0.909	0.659	0.250	0.527	0.399	0.128
≥ 2	0.622	0.349	0.273	0.492	0.301	0.191
≥ 3	0.327	0.145	0.182	0.350	0.181	0.169
≥ 4	0.123	0.043	0.080	0.182	0.077	0.105
≥ 5	0.025	0.004	0.020	0.055	0.017	0.038

Diabetic-level hyperglycemia	ATP III/AHA/NHLBI			IDF		
Cut-off point	Sensitivity	1-specificity	Youden index	Sensitivity	1-specificity	Youden index
≥ 0	1.000	1.000	0.000	1.000	1.000	0.000
≥ 1	0.990	0.778	0.212	0.630	0.453	0.177
≥ 2	0.894	0.458	0.436	0.627	0.384	0.243
≥ 3	0.626	0.205	0.421	0.573	0.242	0.330
≥ 4	0.306	0.063	0.243	0.395	0.107	0.288
≥ 5	0.077	0.009	0.069	0.165	0.024	0.142

*baPWV* brachial-ankle pulse wave velocity, *MetS* metabolic syndrome

The optimal cutoff point of the ATP III/AHA/NHLBI-MetS score to correlate with diabetic-level hyperglycemia was more than or equal to two, with a better sensitivity from 62.6 to 89.4% (Table 2). Differently from the form, the optimal cutoff point of the IDF-MetS score was more than or equal to three, which was equivalent to the current definitions with a sensitivity of 57.3%.

## Discussion

To the best of our knowledge, this is the first study to compare, through AUC–ROC, the performance of ATP III/AHA/NHLBI- and IDF-MetS scores to assess the risks of atherosclerosis-related arterial stiffness and diabetes in a general population. In the 26,735 enrolled subjects, 6633 (24.8%) were classified as having MetS on the basis of the ATP III/AHA/NHLBI criteria, compared to 7388 (27.6%) on the basis of the IDF criteria. The stepwise raises in the baPWV and prevalence of diabetic-level hyperglycemia corresponded significantly to both MetS scores, except for a score of 0 in the IDF-MetS, indicating no abdominal obesity. The performance of the ATP III/AHA/NHLBI-MetS score for a higher baPWV and diabetic-level hyperglycemia was superior to the IDF-MetS score. The optimal cutoff points of the ATP III/AHA/NHLBI-MetS score were more than or equal to two, with a batter sensitivity from 32.7 to 62.2% to correlate with a higher baPWV and from 62.6 to 89.4% to correlate with diabetic-level hyperglycemia.

In the enrolled subjects, 24.8 and 27.6% were classified as having MetS on the basis of the ATP III/AHA/NHLBI and IDF criteria, respectively. Retrospectively, a study from Taiwan in 2002 reported a value of 14.3% with the IDF-MetS [22], almost half of this study. Compared with other Asian countries, the prevalence of the ATP III/AHA/NHLBI-MetS in this study was even more than 13.9% reported in a study from China in 2000–01 [23] and 16.7% in a study from Hong Kong in 1994–96 [24], although the unadjusted ATP III criteria of a fasting glucose level more than or equal to 6.1 mmol/L was used. Accordingly, it was believed that the raising prevalence of the MetS, in particular its approximate twofold increase in Taiwan, mirrors a broadening WC worldwide as the consequence of hypernutrition, a sedentary lifestyle, and sequential abdominal fat accumulation [2, 5]. In addition, a study conducted in the United States from 1999 to 2002 reported that the ATP III/AHA/NHLBI and IDF criteria identified 34.5 and 39.0% [25], respectively, and the lower rate with the ATP III/AHA/NHLBI-MetS score is similar to our results. The lower WC cutoff value of the IDF-MetS may have contributed to this result.

We also showed that stepwise raises in both MetS scores corresponded to the baPWV and prevalence of diabetic-level hyperglycemia, as they were highly correlated with the joint pathophysiology of insulin resistance and represent groups of risk predictors of diabetes and ASCVD [5]. In our previous study, stepwise raises in the MetS-associated risk score, containing a more component of high-sensitivity C-reactive protein, also corresponded to the baPWV in a population without or with chronic kidney disease, and it persisted as a strong determinant of baPWV in the presence of chronic kidney disease or not [19]. In another of our previous studies, stepwise raises in the MetS-associated risk score still corresponded to baPWV before and after fifty in both genders, while it even persisted as a powerful determinant of baPWV only after 50 years old [20]. The conclusions of the above-mentioned studies are compatible with the current study with regards to the significance of the MetS score.

Since these different MetS definitions were established for more than 10 years, differences in sex-specific characteristics and prevalence of the ATP III/AHA/NHLBI- and IDF-MetS among the same population have been frequently published [8, 25]. However, objective comparisons of both MetS definitions to predict clinical outcomes are less common. In addition, for the MetS patients, the prediction of the risk of outcomes according to the definition of MetS is one of most important.

In this study, we showed that through a holistic approach, the performance of the ATP III/AHA/NHLBI-MetS score for the risks of atherosclerosis-related arterial stiffness and diabetes was superior to the IDF-MetS score for Asians. Considerable individual variation has been reported in the clinical pattern of metabolic factors in patients with insulin resistance or obesity [26]. This means that the various expressions of metabolic factors originate from the interactions of genetic factors and environmental exposure [9].

In a cross-sectional survey, Hwang et al. reported that since the IDF-MetS criteria failed to identify non-obese subjects with hypertension, diabetes and dyslipidemia, the ATP III-MetS definition was better than the IDF-MetS definition to detect subjects at a higher risk of ASCVD [22]. This is similar to our finding, however their study lacked an objective and direct comparison of the two MetS definitions. Another previous study used the Framingham score calculation and found a higher risk of ASCVD with the ATP III/AHA/NHLBI-MetS than the IDF-MetS (11.3% vs. 6.1%) [27]. This finding is also similar to ours, however the previous study included further information such as smoking habit beyond MetS itself and lacked a comparison of the risk of diabetes. A recent cross-sectional study enrolling a Chinese elderly population also reported that the updated ATPIII-MetS criteria was a better definition than the IDF-MetS criteria for screening high-risk subjects of ischemic stroke (OR 1.64 vs. 1.55) [28].

By contrast, in the prospective San Antonio Heart Study with 7.4 years of follow-up, the ATP III/AHA/NHLBI and IDF criteria were both associated with an increased but similar ratio of incident CVD and diabetes [29]. In the Framingham Offspring Study with 11 years of follow-up, the ATP III/AHA/NHLBI and IDF criteria both identified a similar increase in the risk of diabetes, while the former may have required insulin resistance to identify an increased risk of ASCVD [30]. In fact, if patients continue to visit an outpatient clinic, longitudinal studies cannot exclude major interference of any additional interventions such as medications, weight reduction or lifestyle modifications during long-term follow-up so that compared to cross-sectional studies, longitudinal studies cannot always detect the natural course and actual clinical outcomes of MetS. This may explain the significant differences in the risk of ASCVD between both MetS definitions in cross-sectional studies [22, 27, 28] but not in longitudinal studies [29, 30].

Interestingly, we also showed that for Asians, the presence of more than or equal to 2 of 5 components of the ATP III/AHA/NHLBI-MetS criteria yielded a better sensitivity than the current criteria. This is a novel finding and very important because increasing obesity caused by a hypernutrition and a sedentary lifestyle [1, 5] is predicted to result in an increase in the global prevalence of diabetes from 6.4 to 7.7%, affecting 439 million adults by 2030 [31]. The MetS is an evolving concept that continues to be data driven and evidence based. For Chinese populations, lowering the threshold of the MetS criteria may remind us of the importance of earlier lifestyle modifications and weight reduction to reduce the further burden of diabetes and ASCVD. However, there is still a need for more evidence.

The study has some limitations. First, some data containing smoking habits, comorbidities, and medications were missed. The information of the questionnaires including family and personal history of chronic diseases and lifestyle optionally are routinely obtained for patients seeking health check. The information may be incomplete, and has not been verified. To avoid misunderstanding we didn’t include the data in our manuscript. However, subjects were mostly asymptomatic and relatively healthy people, who needed to have their health status checked in a self-paid Health Care Center, but different from the National Health Insurance Program in Taiwan [16, 19, 20]. Hence, we believed that the subjects from the Health Care Center are relatively healthy populations. Moreover, the examinations were done after an overnight fast, indicating no consumption of any medications or food for 12 h. At last, samples may have originated from a group of certain wealth and lifestyle that do not exactly represent the whole population of Taiwan. However, due to high accessibility of our Heath Care Center service and a large sample size, the research is believed to be adequate for general representations.

Second, we were unsure as to the appropriate WC cutoff value of the ATP III/AHA/NHLBI-MetS in our Chinese population. However, in the published literature, the same WC cutoff value has been applied in other Asian nations including China [23] and Hong Kong [24]. Third, it was hard to make clear for the associations of the multiple MetS definitions and their outcomes behind our novel conclusions through a holistic approach since this was not a prospective and longitudinal randomized controlled trial. However, this cross-sectional study may identify more objectively the risks of further unfavorable MetS outcomes just now, compared to longitudinal trials, since we minimized disturbances of any supplementary treatments or unexpected changes of physical conditions during long-term follow up.

## Conclusions

This is the first study to demonstrate that through a holistic approach, the performance of the ATP III/AHA/NHLBI-MetS score for the risks of atherosclerosis-related arterial stiffness and diabetes was superior to the IDF-MetS score for Asians. Our novel findings also supported that for Asians, the presence of at least two of five components of the ATP III/AHA/NHLBI-MetS was better than the current criteria, with an improved sensitivity. Through a holistic approach, further prospective and longitudinal randomized controlled studies are required to compare the clinical outcomes between the different MetS definitions.

## Abbreviations

ABI: ankle-brachial index
ASCVD: atherosclerotic cardiovascular disease
ATP III/AHA/NHLBI: Adult Treatment Panel III by the American Heart Association and the National Heart, Lung, and Blood Institute
AUC–ROC: areas under receiver operator characteristic curves
baPWV: brachial-ankle pulse wave velocity
BP: blood pressure
HDL-C: high-density lipoprotein cholesterol
IDF: International Diabetes Federation
MetS: metabolic syndrome
SBP: systolic blood pressure
WC: waist circumference

## References

[1] Collaborators TGO (2017). Health effects of overweight and obesity in 195 countries over 25 years. N Engl J Med.

[2] Grundy SM (2008). Metabolic syndrome pandemic. Arterioscler Thromb Vasc Biol.

[3] Reaven GM (1988). Role of insulin resistance in human disease. Diabetes.

[4] Grundy SM, Brewer HB, Cleeman JI, Smith SC, Lenfant C (2004). Definition of metabolic syndrome: report of the National Heart, Lung, and Blood Institute/American Heart Association conference on scientific issues related to definition. Arterioscler Thromb Vasc Biol.

[5] Cornier MA, Dabelea D, Hernandez TL, Lindstrom RC, Steig AJ, Stob NR, Van Pelt RE, Wang H, Eckel RH (2008). The metabolic syndrome. Endocr Rev.

[6] Eckel RH, Alberti KG, Grundy SM, Zimmet PZ (2010). The metabolic syndrome. Lancet.

[7] Grundy SM, Brewer HB, Cleeman JI, Smith SC, Lenfant C, American Heart A, National Heart L, Blood I (2004). Definition of metabolic syndrome: report of the National Heart, Lung, and Blood Institute/American Heart Association conference on scientific issues related to definition. Circulation.

[8] Delavari A, Forouzanfar MH, Alikhani S, Sharifian A, Kelishadi R (2009). First nationwide study of the prevalence of the metabolic syndrome and optimal cutoff points of waist circumference in the Middle East: the national survey of risk factors for noncommunicable diseases of Iran. Diabetes Care.

[9] Alberti KG, Eckel RH, Grundy SM, Zimmet PZ, Cleeman JI, Donato KA, Fruchart JC, James WP, Loria CM, Smith SC, International Diabetes Federation Task Force on E, Prevention, Hational Heart L, Blood I, American Heart A, World Heart F, International Atherosclerosis S, International Association for the Study of O (2009). Harmonizing the metabolic syndrome: a joint interim statement of the International Diabetes Federation Task Force on Epidemiology and Prevention; National Heart, Lung, and Blood Institute; American Heart Association; World Heart Federation; International Atherosclerosis Society; and International Association for the Study of Obesity. Circulation.

[10] Grundy SM, Cleeman JI, Daniels SR, Donato KA, Eckel RH, Franklin BA, Gordon DJ, Krauss RM, Savage PJ, Smith SC, Spertus JA, Costa F (2005). Diagnosis and management of the metabolic syndrome: an American Heart Association/National Heart, Lung, and Blood Institute Scientific Statement. Circulation.

[11] Alberti KGM, Zimmet P, Shaw J (2005). The metabolic syndrome—a new worldwide definition. Lancet.

[12] Hallajzadeh J, Safiri S, Mansournia MA, Khoramdad M, Izadi N, Almasi-Hashiani A, Pakzad R, Ayubi E, Sullman MJ, Karamzad N (2017). Metabolic syndrome and its components among rheumatoid arthritis patients: a comprehensive updated systematic review and meta-analysis. PLoS ONE.

[13] Imanishi R, Seto S, Toda G, Yoshida M, Ohtsuru A, Koide Y, Baba T, Yano K (2004). High brachial-ankle pulse wave velocity is an independent predictor of the presence of coronary artery disease in men. Hypertens Res.

[14] Turin TC, Kita Y, Rumana N, Takashima N, Kadota A, Matsui K, Sugihara H, Morita Y, Nakamura Y, Miura K, Ueshima H (2010). Brachial-ankle pulse wave velocity predicts all-cause mortality in the general population: findings from the Takashima study, Japan. Hypertens Res.

[15] Cruickshank K (2002). Aortic pulse-wave velocity and its relationship to mortality in diabetes and glucose intolerance: an integrated index of vascular function?. Circulation.

[16] Tsai SS, Lin YS, Chen ST, Chu PH (2018). Metabolic syndrome positively correlates with the risks of atherosclerosis and diabetes in a Chinese population. Eur J Intern Med.

[17] Chang J-B, Chu N-F, Wang S-C, Wu D-M (2007). Metabolic syndrome and its components in relation to brachial-ankle pulse wave velocity in middle-aged Taiwanese males. Open Med..

[18] American Diabetes A (2017). 2. Classification and diagnosis of diabetes. Diabetes Care.

[19] Tsai SS, Lin YS, Lin CP, Hwang JS, Wu LS, Chu PH (2015). Metabolic syndrome-associated risk factors and high-sensitivity C-reactive protein independently predict arterial stiffness in 9903 subjects with and without chronic kidney disease. Medicine..

[20] Tsai S-S, Lin Y-S, Hwang J-S, Chu P-H (2017). Vital roles of age and metabolic syndrome-associated risk factors in sex-specific arterial stiffness across nearly lifelong ages: possible implication of menopause and andropause. Atherosclerosis.

[21] Youden WJ (1950). Index for rating diagnostic tests. Cancer.

[22] Hwang L-C, Bai C-H, Chen C-J (2006). Prevalence of obesity and metabolic syndrome in Taiwan. J Formos Med Assoc.

[23] Gu D, Reynolds K, Wu X, Chen J, Duan X, Reynolds RF, Whelton PK, He J (2005). Prevalence of the metabolic syndrome and overweight among adults in China. Lancet.

[24] Thomas GN, Ho SY, Janus ED, Lam KS, Hedley AJ, Lam TH, Hong Kong Cardiovascular Risk Factor Prevalence Study Steering C (2005). The US National Cholesterol Education Programme Adult Treatment Panel III (NCEP ATP III) prevalence of the metabolic syndrome in a Chinese population. Diabetes Res Clin Pract.

[25] Ford ES (2005). Prevalence of the metabolic syndrome defined by the International Diabetes Federation among adults in the US. Diabetes Care.

[26] Okosun IS, Liao Y, Rotimi CN, Prewitt TE, Cooper RS (2000). Abdominal adiposity and clustering of multiple metabolic syndrome in White, Black and Hispanic Americans. Ann Epidemiol..

[27] Athyros VG, Ganotakis ES, Elisaf M, Mikhailidis DP (2005). The prevalence of the metabolic syndrome using the National Cholesterol Educational Program and International Diabetes Federation definitions. Curr Med Res Opin.

[28] Liu Qian, Li Yan-xun, Zhi-hao Hu, Jiang Xiao-yan, Li Shu-juan, Wang Xiao-feng (2018). Comparing associations of different metabolic syndrome definitions with ischemic stroke in Chinese elderly population. Eur J Intern Med..

[29] Lorenzo C, Williams K, Hunt KJ, Haffner SM (2007). The National Cholesterol Education Program-Adult Treatment Panel III, International Diabetes Federation, and World Health Organization definitions of the metabolic syndrome as predictors of incident cardiovascular disease and diabetes. Diabetes Care.

[30] Meigs JB, Rutter MK, Sullivan LM, Fox CS, D’agostino RB, Wilson PW (2007). Impact of insulin resistance on risk of type 2 diabetes and cardiovascular disease in people with metabolic syndrome. Diabetes Care.

[31] Shaw JE, Sicree RA, Zimmet PZ (2010). Global estimates of the prevalence of diabetes for 2010 and 2030. Diabetes Res Clin Pract.

